# Quantitative inheritance of volatile pheromones and darcin and their interaction in olfactory preferences of female mice

**DOI:** 10.1038/s41598-017-02259-1

**Published:** 2017-05-18

**Authors:** Ying-Juan Liu, Hui-Fen Guo, Jian-Xu Zhang, Yao-Hua Zhang

**Affiliations:** 1 State Key Laboratory of Integrated Management of Pest Insects and Rodents in Agriculture, Institute of Zoology, Chinese Academy of Sciences, Beichen West Road 1-5, Chaoyang District, Beijing, 100101 China; 2 School of Life Science and Technology, Nanyang Normal University, 1638 Wolong Road Nanyang, Henan, 473061 China

## Abstract

In this study, we examined how urine-borne volatile compounds (UVCs) and darcin of male mice are inherited from parents and interact to modulate the olfactory preferences of females using two inbred strains of mice, C57Bl/6 (C57) and BALB/c (BALB), and their reciprocal hybrids (BC = BALB♀× C57♂; CB = C57♀ × BALB♂). Chemical analysis revealed that the UVCs of C57BL/6 males were quantitatively distinguishable from those of BALB/c males. Darcin was detected in C57 urine, but not in BALB urine. The levels of UVCs and darcin in both BC and CB were intermediate between those of C57 and BALB. Behaviourally, C57 females consistently preferred BALB male urine over C57 or CB males despite that there are trace amounts of darcin in BALB urine. However, the preference for BALB urine disappeared in contact two-choice tests of BALB vs. BC pairs, and restored when recombinant darcin was added to BALB male urine. Our results suggested that both UVCs and darcin in male mice are quantitatively inherited and interact to affect the olfactory preferences of females.

## Introduction

Female olfactory preference for indirect genetic benefits has been explained by several mechanisms such as genetic compatibility, good genes, Fisherian sexy son and genetic heterozygosity in animals^1–3^. The physical and chemical signals emitted by males can indicate these genetic conditions and thus are used by females for mate assessment and choice in animals^1^. Chemosensory signals consisting of volatile and nonvolatile compounds are regarded as the most ancient and widespread form in sexual communication across almost all animal taxa^4,5^.

Chemical analysis on urine-borne volatile compounds (UVCs) in mice has mainly focused on the chemical characterization of sex pheromones. In mice, deposited urine bears volatiles derived from bladder urine metabolites and preputial gland secretion (PGS). In particular, urine-metabolized 6-hydroxy-6-methyl-3-heptanone (HMH), R,R-3,4-dehydro-exo-brevicomin (DHB), (S)-2-sec-butyl-4,5-dihydrothiazole (SBT), PGS-derived *E*-β-farnesene, *E*,*E*-α-farnesene, hexadecanol and hexadecyl acetate have been shown to be male pheromones involved in female attraction, intermale aggression, male dominance-submission and female puberty acceleration^6,7^. Some studies have also demonstrated that UVCs contribute to genetic kin recognition and inbreeding avoidance in rodents^8–10^.

On the other hand, increasing evidence suggests that the nonvolatile MUPs (major urine proteins) can also function as protein pheromones as well as transporters and stabilizers of volatile pheromones in rodents^11,12^. MUPs, which have a molecular weight of approximately 18–20 kDa, belong to the lipocalin family and are abundant in rodents. They are mainly synthesized in the liver and excreted in urine^13^. MUPs bind small volatile signal ligands with its β-barrel structures, slowly releasing them and thus greatly prolonging the lifetime of these signals^6,14^. The MUP family proteins are encoded by 21 genes located on chromosome 4 in mice (with >5% sequence identity) and have high sequence diversity^14^. It has been demonstrated with mice that male MUPs are highly polymorphic and can convey olfactory information used by choosy females for genetic heterozygosity assessment, individual discrimination, kin recognition and inbreeding avoidance^15–17^.

Darcin, a male-specific MUP, acts as a nonvolatile protein sex pheromone to attract females, but other tested MUPs do not have the same attractive qualities in mice^18^. Darcin is also responsible for binding most SBT, a male-specific volatile pheromone component^19^. Due to its faster migration on SDS/PAGE compared to major MUPs, darcin is easily separated from other MUPs^20^. In addition to sexual attraction properties, darcin had also been reported to rapidly induce conditioned place preference in females and stimulates hippocampal neurogenesis^12,18,21^.

Although both UVCs and darcin might serve as the scent signal mediating sexual attraction in mice, the relative importance of these two kinds of chemicals is still in debate. Many studies have indicated that the innate attractive sexual pheromones are non-volatile chemicals, and UVCs generally elicit acquired attractive properties when repeatedly associated with these non-volatile compounds, or more accurately—darcin^18,22–25^; however there are more than a few studies indicating that some UVCs are innately attractive to female mice, even when naïve females that have never previously encountered the adult male scent were used in the experiments^26–28^. Actually, increasing evidence indicated that the attraction to urinary odors in rodents would most likely involve blend recognition of both volatile and nonvolatile chemicals, which requiring the collaboration of both main olfactory systems and vomeronasal systems^7,26^.

Here, we investigate the mechanism underlying female olfactory preferences with the two inbred mice strains C57BL/6 (C57) and BALB/c (BALB) mice and their reciprocal hybrids as model animals. Like all inbred strains, their intra-strain members are almost identical in both genotype and phenotype and thus have been widely used as genetically standardized animals to study kin recognition and mate choice^3,29^. BALB males express extremely low level of darcin, thus could be used as an ideal natural mutant strain^18^. We first investigated the inherited characters of UVCs and darcin in the F1 generation of the reciprocal hybrids, and then the attractive properties of volatile and non-volatile stimuli derived from the different strains of male mice were evaluated by using non-contact and contact two-choice tests, respectively. To simplify the comparisons, urine derived from BALB males was selected as an internal control, i.e., the attractiveness of all urine samples were compared to BALB urine, and C57 females served as the subjects. In order to further investigate the effect of darcin in this process, different concentrations of recombinant darcin were added in the donor urine. Our studies may help to reveal the components of sexual attraction in mice, which is the initial step of mate choice between the sexes.

## Methods

### Experimental animals

Thirty-two individual from each strain of BALB and C57 mice (sixteen of each sex) were used as scent donors, and 128 C57 females in estrus were used as odour recipients. The animals were purchased from Weitong-Lihua Experimental Animal Company (Beijing, China) and kept in 14 L: 10 D reverse light cycle (light on at 19:00) at room temperature (23 ± 2 °C). The animals were housed on wood shaving in plastic cages (27 × 12 × 17 cm), and standard mouse chow and water were provided ad libitum. All male mice were housed individually and female mice were housed in groups of four. The animal handling procedure complied with the institutional guidelines for animal use and care at the Institute of Zoology, the Chinese Academy of Sciences. Ethical approval was obtained from the Institutional Ethics Committee of the Institute of Zoology, Chinese Academy of Sciences.

### Cross-breeding procedure

The F1 hybrids were obtained by reciprocally crossing two inbred strains. At approximately 18 weeks of age, BALB females were cross-mated with C57 males and vice versa (named BC and CB, respectively: the maternal strain written first). Intra-strain F1 generations were named BB (BALB♀ × BALB♂) and CC (C57♀ × C57♂), accordingly. Once females were obviously pregnant, they were kept separately. At 4 weeks of age, the pups were weaned, females were grouped in same-sex siblings (2–5 animals per cage), and males were individually housed.

### Sample collection

At 12 weeks of age, samples of urine used in the following behavioural and chemical tests were collected in a clean mouse cage with a wire grid floor. Upon donor urination, the urine was immediately absorbed using a disposable glass capillary and transferred to a vial kept on ice. The mice were then decapitated and their paired preputial glands were immediately dissected and weighted. PGS were then collected in a clear vial by squeezing the glands. All urine and PGS samples were kept at −20 °C until further gas chromatography-mass spectrometry (GC-MS) and protein analysis.

### GC-MS analysis

Sample extractions before GC-MS were conducted as previously described^30,31^. Briefly, each urine and PGS sample was thawed at room temperature and mixed with dichloromethane. The mixture was then thoroughly shaken and stored at 4 °C for 24 h. The lower (dichloromethane) phase of the separated mixture, which contained extracted hydrophobic compounds, was transferred to a new vial and stored at −20 °C until use.

**Figure 1:**
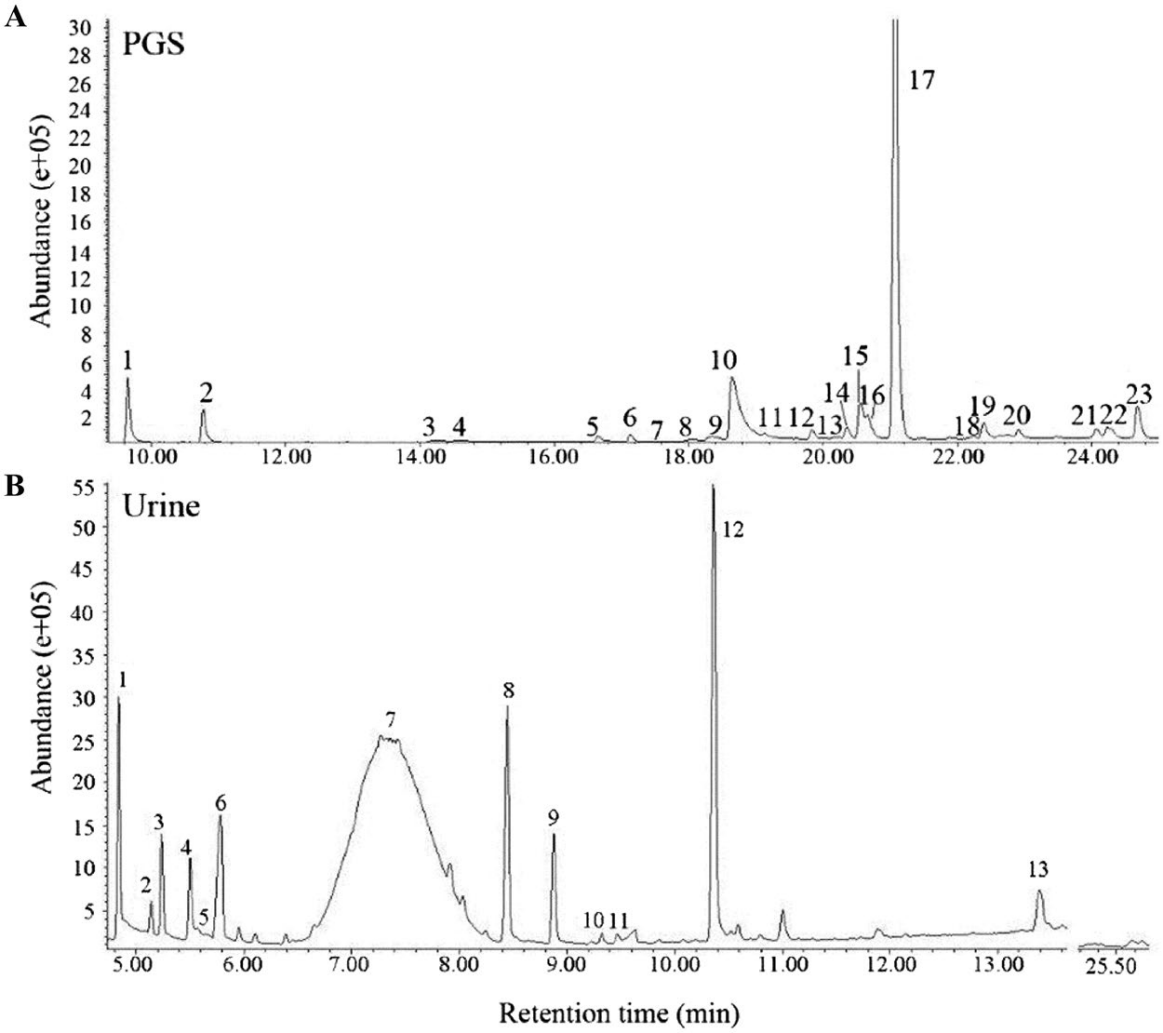
Representative gas chromatogram of PGS (top panel) and urine (bottom panel) from male mice. The numbers used to label GC peaks correspond to the peak numbers in Tables 1 and 2. PGS: preputial gland secretion.

For GC-MS, an Agilent Technologies Network 6890 N GC system combined with a 5973 Mass Selective Detector with the NIST 2002 library were used described previously^31^. The GC was equipped with an HP5MS capillary column (30 m long × 0.25 mm i.d. × 0.25 μm film thickness). The carrier gas was helium at a flow rate of 1.0 mL/min. The injector temperature was 230 °C. The oven temperature program was set as follows: from 100 °C to 230 °C (26 min) at 5 °C/min and then to 280 °C at 10 °C/min, holding for 15 min (for PGS samples); from 50 °C to 150 °C (20 min) at 5 °C/min and then to 230 °C (8 min) at 10 °C/min, holding for 2 min (for urine samples). The post run lasted for 15 min at 280 °C to clean the column. Electron impact ionization was applied at 70 eV, and the transfer line temperature was maintained at 280 °C. The mass range scanned was from 30 to 350 amu. The PGS (1 μL) and urine (4 μL) extractions were injected manually in a split (10:1) and a splitless mode, respectively. The representative gas chromatograms of the volatile compounds from urine and PGS are presented in Fig. 1.

### SDS-PAGE

SDS-PAGE was performed as previously described^20,32^. Urine samples to be analysed were diluted 4 times and mixed with 5x sample buffer [250 mM Tris-HCl at pH 6.8, 10% (w/v) SDS, 0.5% (w/v) bromophenol blue, 50% (v/v) glycerol, and 5% (v/v) β-mercaptoethanol]. Then 4-μL mixed samples were fractionated by SDS-PAGE at a constant voltage of 130 V on 15% gels. Based on the atypical mobility of daricn on SDS-PAGE, the level of darcin in urine was assayed by Coomassie brilliant blue staining of the SDS-PAGE gels. The intensities of the bands were quantified using the Image J program.

### Expression and purification of recombinant darcin

Purification of recombinant darcin (Mup20) produced in *E*. *coli* was performed as published^18^. The cDNA for darcin (Mup20, GenBank accession number Q5FW60.1) was *de novo* synthetized and cloned into pET28a via NcoI and XhoI restriction enzyme sites. The constructed vector was transformed into *E*. *coli* BL21(λ)DE3 competent cells. The recombinant protein was purified via Ni-NTA agarose column (QIAGEN, Germany), following the manufacturer’s recommendations. Purified recombinant darcin was pooled and dialyzed against 1 × PBS buffer.

To determine the approximate concentration of darcin in male urine, the mixed urine samples derived from four males of each line were compared with gradient diluents of purified recombinant darcin on the SDS-PAGE gel.

### Behavioural tests

The responses of mice to urine samples were tested in their home cages under a red light during the dark phase as described previously^10^. In noncontact two-choice tests, 2 μL of urine sample was injected into a disposable glass capillary tube (i.d. 1.1–1.2 mm, o.d. 1.3–1.4 mm, 15 cm length), which was then sealed with odourless gum at one end. The sample remained inside the capillary tube, 1 cm from the capillary tip, so that the mice could not come into direct contact with the sample, and only volatiles were accessible to the subjects. The sample-containing ends were then presented to the test mice.

In contact two-choice tests, 5 μL of each urine sample was randomly smeared onto one of two ends (2.5 cm × 1.5 cm) of a glass slide (2.5 cm × 7.6 cm) and then the slide was vertically fixed on the cage lid, therefore, both volatile and non-volatile chemicals were accessible to the subjects. In both contact and noncontact two-choice tests, the total time that the subject spent actively sniffing and contacting the tips or smeared ends within 3 min were recorded by an observer who was blind to the experimental setup.

### Data analysis and statistics

We first examined the distribution of all raw data using the Kolmogorov–Smirnov test in SPSS for Windows. For normally distributed data, one-way analysis of variance (ANOVA) followed by least significant difference (LSD) post hoc *t*-tests, were conducted to analyse GC-MS data, paired-samples *t*-tests were used to assess behavioural data, and Student’s t-tests were employed to analyse SDS-PAGE data. For non-normally distributed data, Kruskal-Wallis *H* with post hoc Mann–Whitney *U* tests were used for GC data and Wilcoxon signed ranks tests for behavioural data. The GC-MS data were also subjected to principal components analysis (PCA) for further variation analysis. All analyses were conducted using SPSS 15.0, and the level of significance was set at α = 0.05 (two-tail).

## Results

### Volatiles abundance in preputial gland and urine

**Figure 2:**
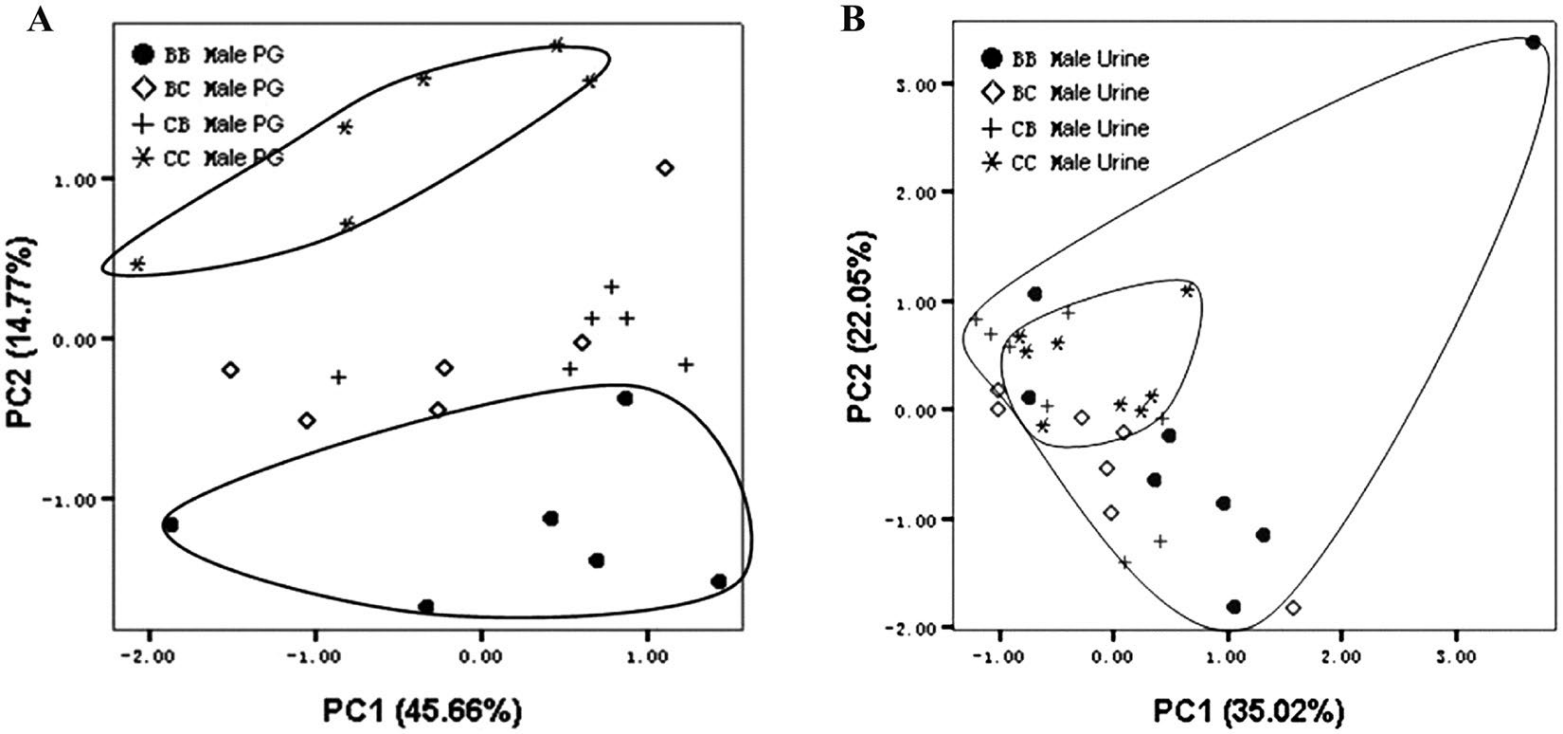
Principle component analysis of PGS (**A**) and urine (**B**) data. Each sample is represented by the initial letters of the strain name; *n* = 6 in each group. BB: BALB/c strain; CC: C57BL/6 strain; BC: hybrid of BALB♀ × C57♂; CB: hybrid of C57♀ × BALB♂; PGS: preputial gland secretion.

In PGS, a total of 23 compounds were detected, 4 compounds (compounds 12: *F*_3,20_ = 5.862, *P* = 0.005; 18: *F*_3,20_ = 19.52, *P* < 0.001; 19: *F*_3,20_ = 19.57, *P* < 0.001; and 21: *H*_3_ = 13.01, *P* = 0.005) varied in relative abundances among the four strains of mice. In detail, all four compounds differed between C57 and BALB males; compounds 12, 18 and 19 differed between C57 and the two reciprocals hybrids; compound 21 differed between BALB and the two reciprocal hybrids; and compound 19 differed between BC and CB (Table 1). PCA plot showed that the two inbred strains were completely separated along the PC2 axis and the hybrids were intermediate between the two inbred strains (PC2, 14.77% of variance, Fig. 2A).

**Table 1:** Relative abundance of preputial gland volatiles in the four groups.

GC Peak	RT min	Compounds	BB	CC	BC	CB
1	9.65	E-β-farnesene	4.95 ± 2.40	3.67 ± 1.85	4.71 ± 2.46	7.02 ± 2.10
2	10.77	E,E-α-farnesene	2.55 ± 1.35	1.95 ± 1.12	2.54 ± 1.51	3.75 ± 1.24
3	14.18	Z-7-Tetradecenol	0.35 ± 0.18	0.37 ± 0.22	0.33 ± 0.20	0.48 ± 0.25
4	14.58	1-Tetradecanol	0.22 ± 0.10	0.18 ± 0.06	0.17 ± 0.09	0.21 ± 0.09
5	16.68	Z-5-Tetradecenol acetate	0.41 ± 0.22	0.79 ± 0.40	0.62 ± 0.38	0.82 ± 0.25
6	17.15	1-Tetradecanol acetate	0.56 ± 0.25	0.71 ± 0.33	0.69 ± 0.33	0.84 ± 0.22
7	17.61	Z-11-Hexadecenol	0.12 ± 0.05	0.10 ± 0.05	0.11 ± 0.06	0.12 ± 0.07
8	17.99	1-Hexadecanol (branched)	0.23 ± 0.08	0.15 ± 0.09	0.15 ± 0.08	0.22 ± 0.06
9	18.29	Z-9-Hexadecenol	0.53 ± 0.26	0.43 ± 0.15	0.37 ± 0.21	0.50 ± 0.20
10	18.68	1-Hexadecanol	13.01 ± 5.35	8.83 ± 1.66	8.24 ± 3.63	10.23 ± 2.94
11	19.16	1-Pentadecanol acetate	0.19 ± 0.05	0.16 ± 0.10	0.17 ± 0.07	0.21 ± 0.09
12	19.86	Z-9-Hexadecenol acetate	0.82 ± 0.23^a^	0.62 ± 0.12^a,b,c^	0.81 ± 0.11^b^	0.97 ± 0.08^c^
13	20.18	1-Heptadecanol (branched)	0.12 ± 0.08	0.06 ± 0.03	0.05 ± 0.02	0.08 ± 0.03
14	20.38	1-Hexadecanol acetate (branched)	1.06 ± 0.28	0.77 ± 0.21	1.00 ± 0.21	1.11 ± 0.14
15	20.58	Isomer of Z-11-Hex decenol acetate	2.39 ± 0.63	3.37 ± 0.97	2.56 ± 0.54	3.00 ± 0.43
16	20.69	Z-11-Hexadecenol acetate	0.52 ± 0.33	0.39 ± 0.22	0.61 ± 0.15	0.53 ± 0.18
17	21.18	1-Hexadecanol acetate	63.83 ± 8.40	69.11 ± 4.74	68.35 ± 6.17	61.79 ± 5.16
18	22.25	1-Heptadecanol acetate (branched)	0.17 ± 0.06^a^	0.48 ± 0.10^a,b,c^	0.20 ± 0.08^b^	0.24 ± 0.06^c^
19	22.41	1-Heptadecanol acetate (branched)	1.70 ± 0.25^a,b^	0.84 ± 0.21^a,c,d^	1.09 ± 0.13^b,d^	1.47 ± 0.21^d^
20	22.93	1-Heptadecanol acetate	0.81 ± 0.20	0.56 ± 0.24	0.73 ± 0.13	0.83 ± 0.16
21	24.08	Dodecyl octanoate	0.88 ± 0.17^a,b,c^	1.46 ± 0.31^a^	1.47 ± 0.25^b^	1.34 ± 0.12^c^
22	24.24	Z-7-Octadecenol acetate	0.58 ± 0.19	0.86 ± 0.52	0.52 ± 0.27	0.61 ± 0.16
23	24.70	Octadecanol acetate	3.99 ± 0.98	4.15 ± 1.15	4.20 ± 0.91	3.64 ± 0.47

Data are presented as mean ± SD, *n* = 6 for each group; compounds were identified using mass library (NIST 2002). ^a–e^Means in a row marked by a same superscript letter show significant differences (*P* < 0.05, using one-way ANOVA with LSD post hoc t-test or Kruskal–Wallis H with post hoc Mann–Whitney *U* test). BB: BALB/c strain; CC: C57BL/6 strain; BC: hybrid of BALB♀ × C57♂; CB: hybrid of C57♀ × BALB♂; RT: retention time.

Of the 13 urine-metabolized volatiles detected by GC-MS, 5 compounds (5: *H*_3_ = 10.77, *P* = 0.013; 6: *H*_3_ = 9.588, *P* = 0.022; 9: *H*_3_ = 10.60, *P* = 0.014; 11: *F*_3,28_ = 5.632, *P* = 0.004 and 13: *F*_3,28_ = 17.08, *P* < 0.001) varied among the four groups (Table 2). In detail, all the five components differed between BALB and C57 mice; compounds 6, 9, 11 and 13 differed between BALB and the two hybrids; compounds 5 and 6 differed between C57 and CB; and compound 5 differed between C57 and BC; and compound 9 differed between the BC and CB. The PCA plot showed no distinction between the hybrid and inbred males (Fig. 2B).

**Table 2:** Relative abundance of urinary volatiles in the four groups.

GC peak	RT min	Compounds	BB	CC	BC	CB
1	4.83	*Z*-5,5-dimethyl-2-ethylidenetetrahydrofuran	2.53 ± 1.47	2.43 ± 0.68	1.95 ± 0.64	2.43 ± 1.19
2	5.14	2-Heptanone	0.40 ± 0.83	0.20 ± 0.30	0.07 ± 0.04	0.11 ± 0.13
3	5.24	*E*-5-Hepten-2-one	0.59 ± 0.44	0.54 ± 0.37	0.39 ± 0.20	0.55 ± 0.35
4	5.50	*E*-5,5-dimethyl-2-ethylidenetetrahydrofuran	0.95 ± 0.52	0.83 ± 0.24	0.70 ± 0.21	0.87 ± 0.41
5	5.55	*Z*-2-pentenyl acetate	0.05 ± 0.12^a^	0.14 ± 0.11^a,b,c^	0.03 ± 0.07^b^	0.01 ± 0.02^c^
6	5.77	Dimethyl sulfone 6-Hydroxy-6-methyl-3-heptanone and	10.5 ± 7.20^a,b,c^	6.10 ± 2.12^a,d^	5.62 ± 3.31^b^	4.42 ± 2.53^c,d^
7	7.43	5,5-dimethyl-2-ethyltetrahydrofuran-2ol	57.7 ± 9.72	68.0 ± 10.3	67.3 ± 12.7	69.0 ± 11.6
8	8.45	Benzyl alcohol	6.34 ± 4.00	4.87 ± 3.59	5.79 ± 2.54	5.83 ± 2.10
9	8.88	*R*,*R*-3,4-Dehydro-exo-brevicomin	6.63 ± 3.36^a,b,c^	2.53 ± 1.53^a^	3.53 ± 2.01^b^	2.18 ± 1.23^b,c^
10	9.34	Acetophenone	0.19 ± 0.12	0.13 ± 0.05	0.23 ± 0.11	0.19 ± 0.06
11	9.48	*O*-Toluidine	0.65 ± 0.30^a,b,c^	0.32 ± 0.25^a^	0.21 ± 0.18^b^	0.26 ± 0.21^c^
12	10.36	(S)-2-*Sec*-butyl-4,5-dihydrothiazole	10.9 ± 6.50	13.2 ± 5.61	13.2 ± 6.99	13.1 ± 8.29
13	13.38	N-Phenyl formanide	2.55 ± 0.89^a,b,c^	0.75 ± 0.51^a^	0.93 ± 0.40^b^	1.04 ± 0.28^c^

Data are presented as mean ± SD, *n* = 8 for each group; compounds were identified using mass library (NIST 2002). ^a–f^Means in a row marked by a same superscript letter show significant differences (*P* < 0.05, using one-way ANOVA with LSD post hoc t-test or Kruskal–Wallis H with post hoc Mann–Whitney *U* test). BB: BALB/c strain; CC: C57BL/6 strain; BC: hybrid of BALB♀ × C57♂; CB: hybrid of C57♀ × BALB♂; RT: retention time.

### MUPs profile in the urine

**Figure 3:**
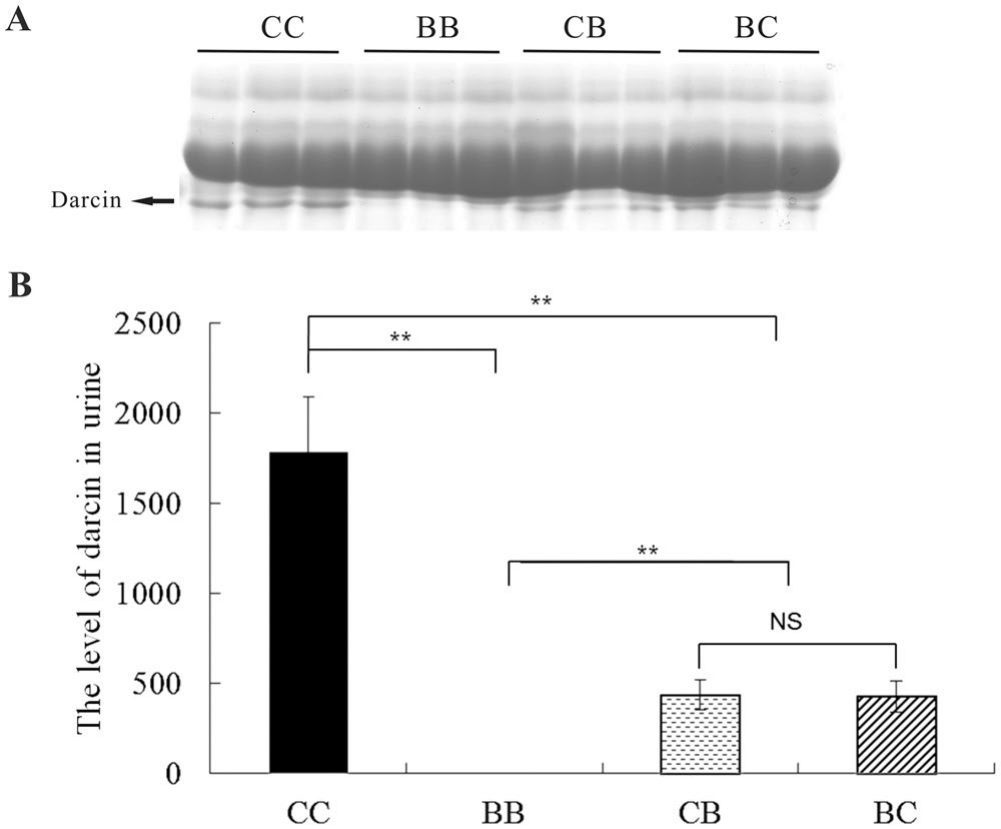
SDS-PAGE of urine from F1 progeny. (**A**) Representative image, darcin is indicated by an arrow; (**B**) Band intensities of darcin from the SDS-PAGE were quantified using the Image J program. The data are represented as mean ± SE, *n = *8 in each group. **Indicates significant difference of values at *P* < 0.01 by Student’s *t* test. BB: BALB/c strain; CC: C57BL/6 strain; BC: hybrid of BALB♀ × C57♂; CB: hybrid of C57♀ × BALB♂; NS: not significant.

Figure 3A shows a representative image of MUPs separated by SDS-PAGE. According to the Image J results, the intensities of MUPs were not different between the four groups. Darcin constituted approximately 10% of the total MUPs in C57, but it was barely detectable in the BALB urine (Fig. 3B), which is consistent with previous studies^18,33^. The darcin levels in BC and CB were intermediate between their parental strains, suggesting that it is quantitative hereditability.

### Olfactory preferences of C57 females

**Figure 4:**
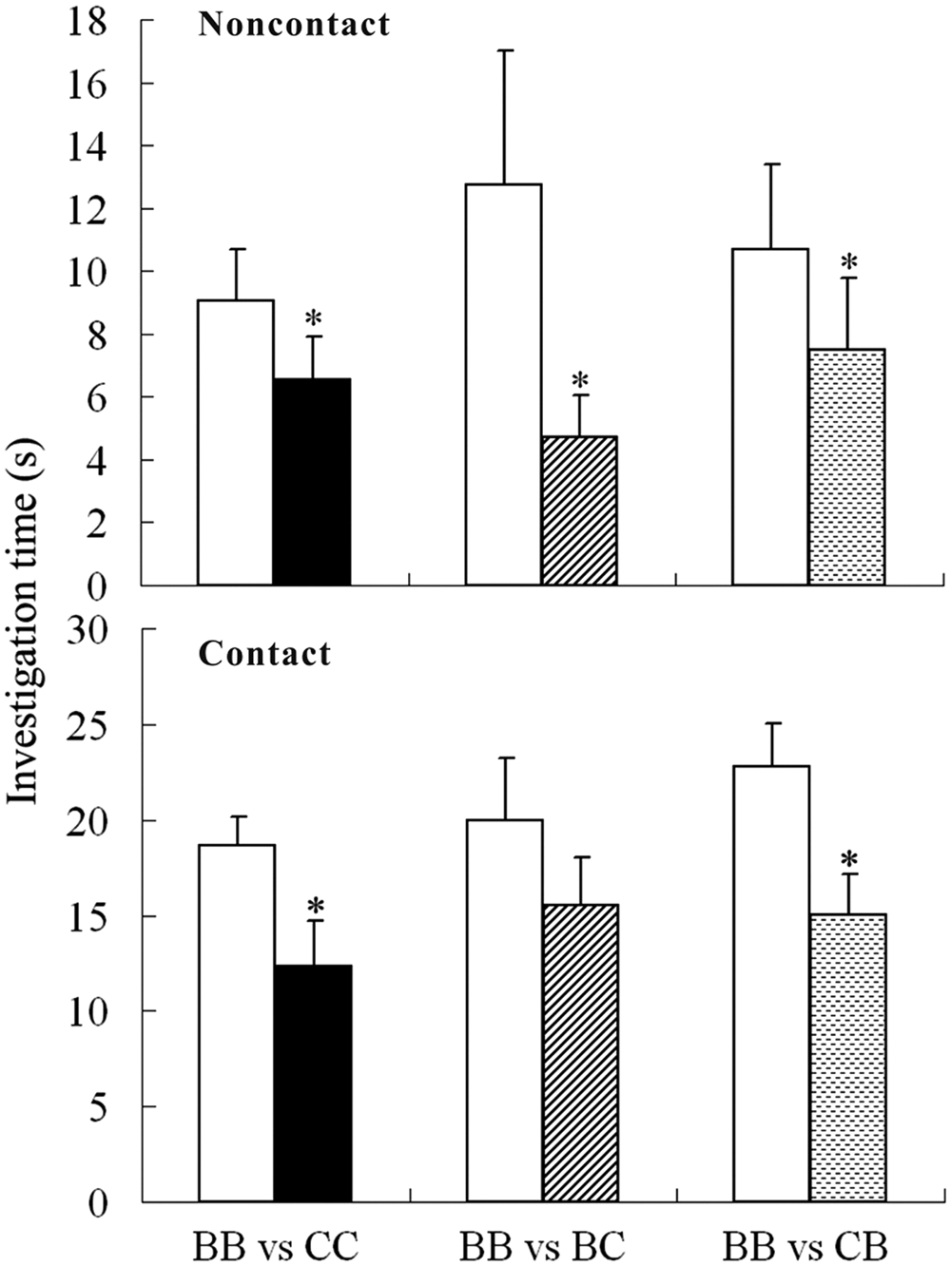
Responses of female C57BL/6 to male urine from different strains. In each test, mice were presented with two urine samples, one from BB and the other from CC, BC or CB mice. *Indicates a significant difference between the investigation times in each trial. The data are presented as the mean ± SE, *n* = 16 in each group. BB: BALB/c strain; CC: C57BL/6 strain; BC: hybrid of BALB♀ × C57♂; CB: hybrid of C57♀ × BALB♂.

In noncontact two-choice tests, C57 females significantly preferred BALB male urine to male urine from C57 or hybrid males (BB vs. CC: *z* = 2.223, *P* = 0.026; BB vs. BC: *z* = 2.395, *P* = 0.017; BB vs. CB: *z* = 2.401, *P* = 0.016). In contact two-choice tests, C57 females also preferred BALB male urines in the BB/C57 and BB/CB paired odours, which suggests that genetic compatibility may account for choice preferences (BB vs. CC: *t* = 2.485, *P* = 0.026; BB vs. CB: *t* = 2.802, *P* = 0.015). However the preference for BALB urine disappeared in the BB vs. BC pairs (BB vs. BC: *t* = 1.063, *P* = 0.305) (Fig. 4).

### Darcin addition affected the olfactory preference of C57 females

**Figure 5:**
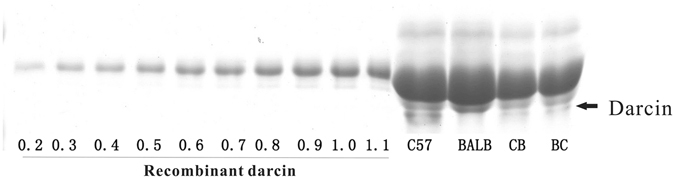
SDS-PAGE of recombinant darcin diluents and urine from C57, BALB, CB and BC male mouse. One microliter sample was used in the analysis. Recombinant darcin was diluted to different concentrations. The value numbers below the band of recombinant darcin refer to the concentration (μg/μL) of recombinant darcin in corresponding diluents. BC: hybrid of BALB♀ × C57♂; CB: hybrid of C57♀ × BALB♂.

**Figure 6:**
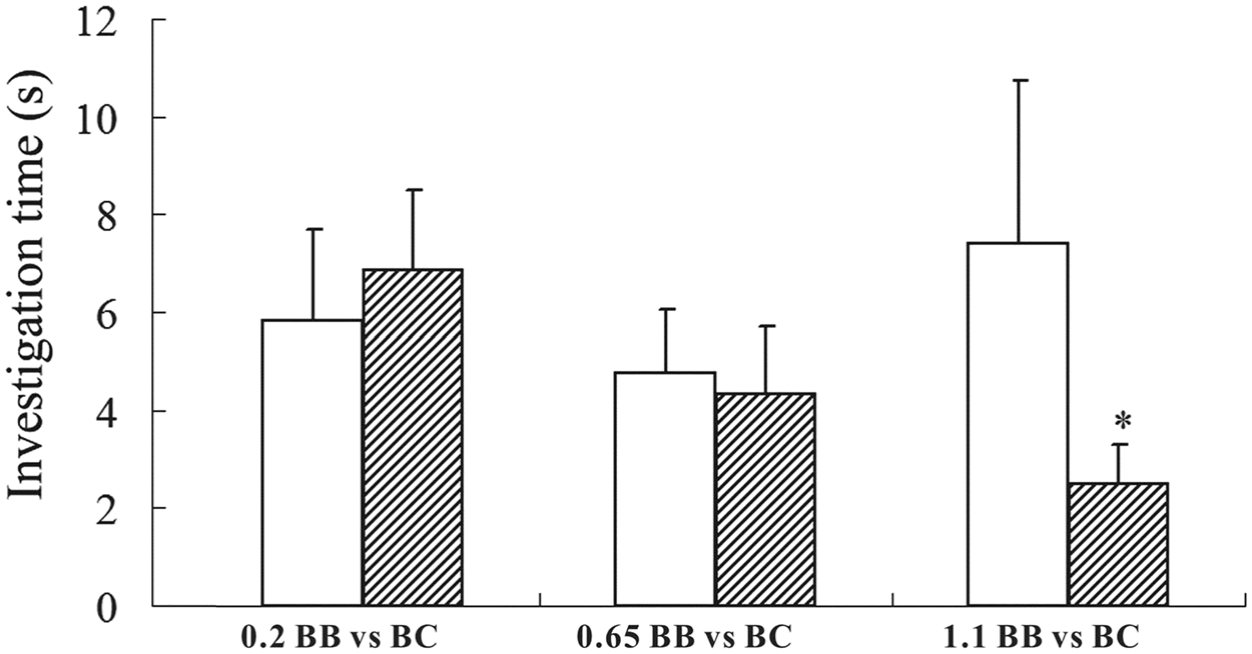
Responses of female C57BL/6 to BC and BB male urine with different levels of recombinant darcin added. In each test, mice were presented with two urine samples from BB (white bars), BC (diagonal hatched bars). Values before BB refer to the concentration of recombinant darcin added (μg/μL). *Indicates a significant (*P* < 0.05) difference between the investigation times in each trial. The data are presented as mean ± SE, *n* = 12 in each group. BB: BALB/c strain; BC: hybrid of BALB♀ × C57♂.

To investigate whether darcin accounted for the disappeared preference for BALB in contact two-choice tests of BALB vs. BC paired urines, different concentrations of recombinant darcin were added to BALB urine. SDS-PAGE results indicated that the urine of BC males contained approximately 0.2 μg/μL of darcin, and C57 male urine had 1.1 μg/μL (Fig. 5). When recombinant darcin was added at a level of 0.2 μg/μL (equal to BC males), C57 females still showed no preference for the treated BALB urine (*z* = 0.280, *P = *0.779, *n = *8). However, when the concentration of added darcin reached 1.1 μg/μL (equal to C57 male), C57 females show higher attraction to the treated BALB urine (*z = *2.033, *P = *0.042, *n = *8) (Fig. 6). As indicated, recombinant darcin show lower mobility than urinary darcin on SDS-PAGE (Fig. 5). The reasons for this are not clear, but it does not affect its biological function in general^18^.

## Discussion

Signals produced by one sex and received by the other play a critical role in mediating social behaviour and reproductive interactions across the animal worlds^7,34,35^. In rodents, chemicals in the urine provide multiple types of information such as sex, age, health status, hormonal levels and genetic background to the conspecifics^3,15,16,36,37^. Consistent with previous studies, our results indicated that UVC were positively correlated with genetic relatedness, which was exhibited by quantitative differences and the degree of separation between the strains in PCA plots. Furthermore, our results also showed that both darcin and UVC were quantitatively inherited from their parents, as indicated by the level of these chemicals in the F1 hybrid being intermediate between the parental generation.

It has been previously reported that darcin elicit innate female sexual attraction, and UVCs only induce conditional olfactory preference in females^12,18,25^. However, the current results demonstrated that despite the fact that BALB male mice had only trace amounts of darcin, they were always preferred by C57 females in both noncontact and contact tests. This, along with previous studies from our group and others^12,26–28,38,39^, indicates that genetic compatibility may play an important role in the initial sexual attraction even in these classic laboratory strain of mice, which have been divorced from the normal pressures of sexual selection in nature for hundreds of generations. Genetic compatibility maybe determined by a blend of chemicals such as UVCs, MUPs, and MHC ligands, which fuction in gestalt or mosaic forms in rodents social life^26,39–41^. In this case, we don’t think one or two particular compounds play a major role in the initial olfactory preferences of female mice.

Strikingly, the volatiles identified in the present study do not correlate with the chemical compounds identified in the urine of Swiss strain male mice^42,43^, in which the majority of compounds are long chain alkanes. It therefore appears that the chemicals involved in chemical communication differ depending on the genetic background. However, we cannot exclude the possibility that the GC system employed (column and film differences) and temperature program settings during the tests may account for this discrepancy. In addition to these factors, food, social status and gut microflora of the subjects has also been demonstrated to affect the odour type^44^. Nevertheless, the male-typical chemicals DHB and SBT were verified in our test. Additionally, the identified chemicals correlated well with previous studies using the same strain of mice^26,45^. These may indicate to a certain degree the validity of our identification in some aspect.

Another interesting finding of our study is that the preferece for BALB odours in BALB/BC paird odours in the non-contact two-choice test disappeared in the contact two-choice test. This is confusing because contact tests, where the subject could access both volatile and non-volatile chemicals, should provide more accurate information to the subjects. The plausible explanation is that the darcin level or heterozygosity of MUPs in BC males might conteract the effects of UVC-associated genetic compatibility in the olfactory preference of the females. Such a “darcin effect” was further supported by the recurrence of olfactory preference after adding recombinant darcin to BALB male urine to a level of 1.1 μg/μL, which corresponded to the concentration of C57 males but greatly exceeded that of BC males.

However, this explanation fails when interpreting BALB/CB paird odors, where C57 invariably prefer BALB urine in both non-contact and contact two-choice test. As indicated, although BC and CB males theoretically share the same complement of genes from C57 and BALB mice, the genetic and epigenetic differences between obverse and reverse crosses, resulting from sex-linked inheritance, linkage disequilibrium, genetic imprinting and maternal effects, might cause some genetic shift between BC and CB males^46,47^. In addition, a tiny genetic modification, such as a single gene mutation, can elicit changes to male UVCs and MUPs, which in turn send genetic messages to choosy females in mice^9,48^. Likewise, odour types were not identical between BC and CB in the current study (compound 19 in Table 1 and compound 9 in Table 2 were different).

Overall, our study indicated that both UVCs and darcin from males are quantitatively inherited and interact to affect the olfactory preferences of females. As indicated, in nature, as is the case for human abilities to recognize individuals based on a variety of visual characteristics, in mice there is likely to be multiple, redundant mechanisms underlying olfactory individuality, e.g., MHC, MUPs, UVCs, social learning, and additional undiscovered mechanisms. We are just starting to understand how different information in male scents is integrated in female olfactory preferences. Our studies are meaningful as we provide novel insight into the mechanisms in sex recognition and mate assessment in rodents.

There are several limitations to this study. First, we only used C57 females as the odour receptor, and it is not clear whether these results apply to other laboratory strains, or more importantly, to wild mice. Second, the role of MUPs were not fully investigated in this study, e.g., what would be the reaction of the female mice if only MUPs were available (devoid of all the adhered volatiles)? All these areas require further study in the future.
