# Author Correction: Quantitative inheritance of volatile pheromones and darcin and their interaction in olfactory preferences of female mice

**DOI:** 10.1038/s41598-018-22515-2

**Published:** 2018-03-06

**Authors:** Ying-Juan Liu, Hui-Fen Guo, Jian-Xu Zhang, Yao-Hua Zhang

**Affiliations:** 1 State Key Laboratory of Integrated Management of Pest Insects and Rodents in Agriculture, Institute of Zoology, Chinese Academy of Sciences, Beichen West Road 1-5, Chaoyang District, Beijing, 100101 China; 2 School of Life Science and Technology, Nanyang Normal University, 1638 Wolong Road Nanyang, Henan, 473061 China

Correction to: *Scientific Reports* 10.1038/s41598-017-02259-1, published online 18 May 2017

This Article contains typographical errors in Table 2.

For GC peak 6, ‘Dimethyl sulfone 6-Hydroxy-6-methyl-3-heptanone and’ should read ‘Dimethyl sulfone’.

In addition, for GC peak 7 ‘5,5-dimethyl-2-ethyltetrahydrofuran-2ol’ should read ‘6-Hydroxy-6-methyl-3-heptanone and 5,5-dimethyl-2-ethyltetrahydrofuran-2ol’.

